# Severe pneumomediastinum in an infant with respiratory syncytial virus infection: A case report

**DOI:** 10.1097/MD.0000000000042038

**Published:** 2025-04-04

**Authors:** Junji Egawa, Makiko Konda, Yusuke Naito, Taichi Kotani, Shota Sonobe, Masahiko Kawawguchi

**Affiliations:** a Department of Anesthesiology, Nara Medical University, Nara, Japan.

**Keywords:** Macklin effect, pneumomediastinum, respiratory syncytial virus, subcutaneous emphysema

## Abstract

**Rationale:**

The respiratory syncytial virus (RSV) is an important cause of bronchiolitis in children, with limited reports of pneumomediastinum as a complication. We report an infant with pneumomediastinum associated with RSV infection requiring intubation.

**Patient concerns:**

A 4-month-old male infant was diagnosed with RSV infection on the 2nd day of symptom onset (coughing and fever). On the 7^th^ day of symptom onset, chest radiography and computed tomography revealed severe pneumomediastinum, and thus, he was transferred to the intensive care unit.

**Diagnosis:**

Severe pneumomediastinum secondary to RSV infection.

**Interventions:**

The patient was intubated, and lung-protective ventilation with muscle relaxants was initiated. After 42 hours of continuous muscle relaxant administration, weaning from mechanical ventilation was started. He was extubated after confirming improved oxygenation and favorable imaging findings.

**Outcomes:**

The infant recovered completely without further worsening of pneumomediastinum.

**Lessons:**

An infant with severe pneumomediastinum requiring intubation during the course of RSV infection was successfully managed with lung-protective ventilation using muscle relaxants.

## 1. Introduction

Acute bronchiolitis due to respiratory syncytial virus (RSV) infection is the leading cause of hospitalization in infants aged under 1 year.^[1]^ Risk factors for increased RSV infection severity include prematurity, infants aged < 6 months in the beginning of RSV season, cystic fibrosis, being male, chronic lung disease, bronchopulmonary dysplasia, congenital heart disease, immunodeficiency, cerebral palsy, neuromuscular system diseases, and Down syndrome.^[2–4]^

Pneumomediastinum is defined as the presence of air in the mediastinum and is diagnosed via chest X-ray or computed tomography (CT) scanning.^[5]^ However, the incidence of pneumomediastinum, including traumatic and iatrogenic causes, in infants over the age of 4 weeks in the intensive care unit (ICU) is very small.^[6]^ Although various causes of pneumomediastinum, such as bronchospasm, cough, respiratory tract infection, valsalva maneuvers (vomiting), foreign body aspiration, trauma, athletic exertion and huffing, and esophageal perforation, have been reported,^[6–10]^ pneumomediastinum associated with pediatric RSV infection is rare, and limited cases have been reported.^[8,11–14]^ All of these patients, except for 1, were successfully treated with supplemental oxygen or noninvasive positive-pressure ventilation. Herein, we report the case of an infant with severe pneumomediastinum associated with RSV infection who required mechanical ventilation under muscle relaxant.

## 2. Case presentation

**Figure 1.**
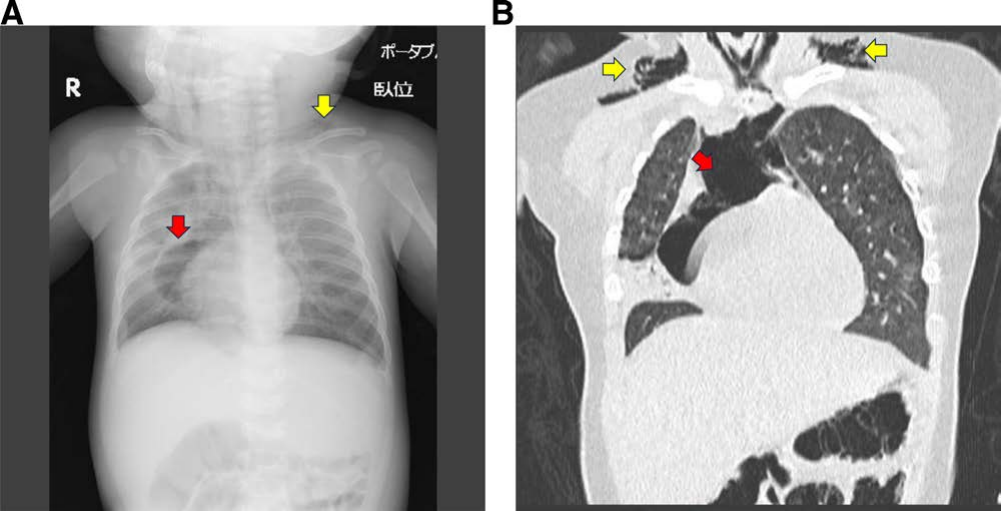
(A) Chest X-ray shows pneumomediastinum (red arrow) and subcutaneous emphysema (yellow arrow). (B) Coronal CT image shows severe pneumomediastinum (red arrow) and subcutaneous emphysema (yellow arrows). CT = computed tomography.

**Figure 2.**
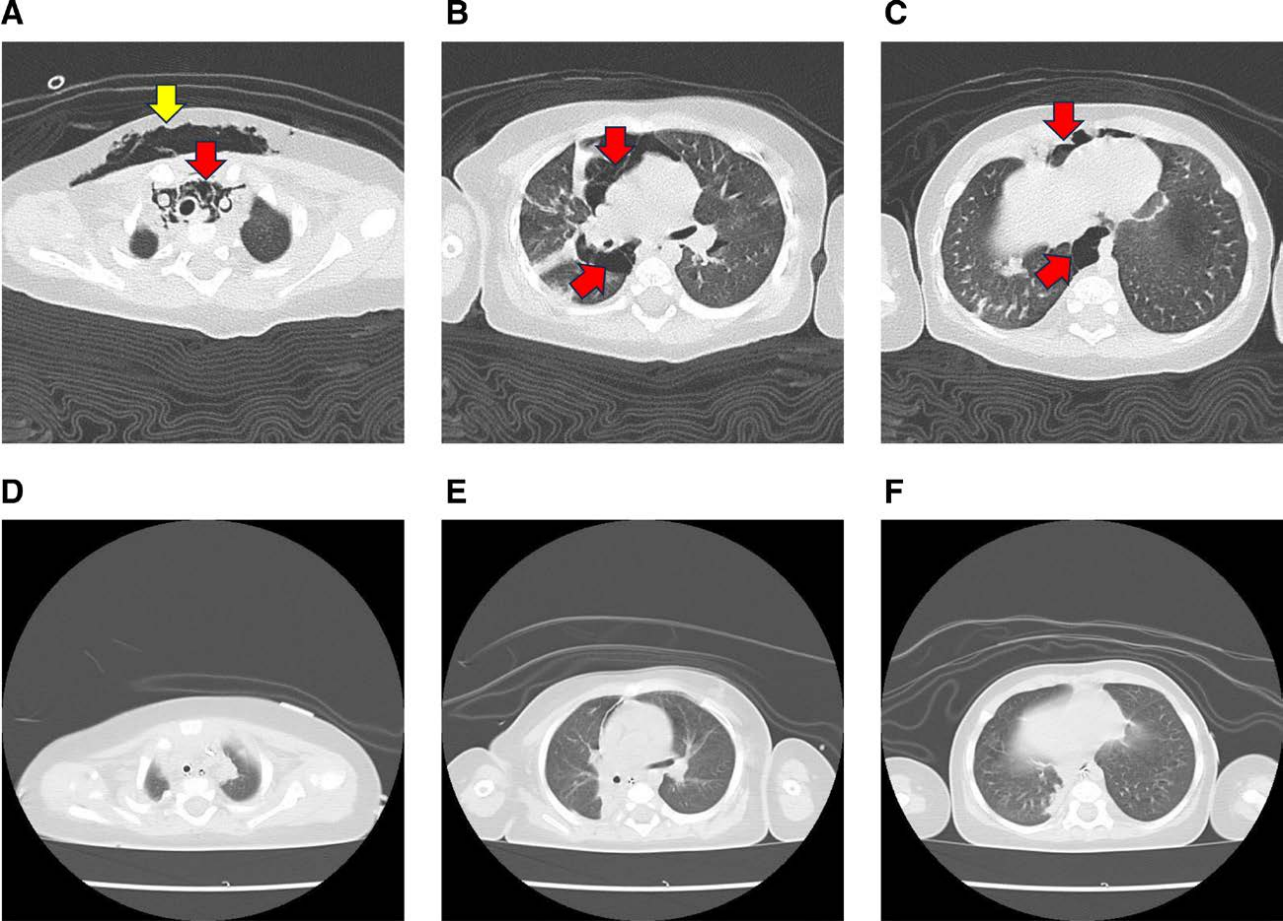
(A–C) Axial CT images on the day of ICU admission showed severe pneumomediastinum (red arrow) and subcutaneous emphysema (yellow arrows). (D–F) Axial CT images on the 5th day of ICU admission demonstrate the improvement of pneumomediastinum and subcutaneous emphysema. CT = computed tomography, ICU = intensive care unit.

A 4-month-old male infant (height, 66 cm; weight, 7.2 kg) was admitted to our hospital with respiratory failure due to pneumomediastinum associated with RSV infection. He was born spontaneously at 39 weeks of gestation, with no postnatal abnormalities. RSV infection was diagnosed by his family doctor on the 2nd day after the onset of cough and fever using an RSV rapid test kit. He developed severe bronchiolitis and was hospitalized at a neighborhood hospital on the 4th day, where he received supplemental oxygen, beta stimulants, and methylprednisolone 2.0 mg/kg/d. Pathogen detection was performed using the BioFire FilmArray Respiratory Panel 2.1 (BioFire Diagnostics, Inc., Salt Lake City). This multiplex real-time PCR assay panel included adenovirus, coronavirus HKU1, coronavirus NL63, coronavirus 229E, coronavirus OC43, human metapneumovirus, human rhinovirus/enterovirus, influenza A, influenza B, parainfluenza virus 1, parainfluenza virus 2, parainfluenza virus 3, parainfluenza virus 4, RSV, and SARS-CoV-2. This panel also includes bacterial pathogens such as Bordetella pertussis, Chlamydia pneumoniae, and Mycoplasma pneumoniae. In addition to PCR testing, sputum cultures were performed to identify potential bacterial pathogens. The PCR panel detected only RSV, and both initial and follow-up sputum cultures were negative for bacterial growth. Additionally, the patient had no documented history of RSV infection, no recurrent respiratory infections, and no underlying conditions predisposing to severe infections. There were no relatives with RSV infection among the patient’s family, and regional outbreaks were not reported in the patient’s area of residence. On the 5th day of symptom onset, high-flow nasal cannula (HFNC) oxygen therapy (15 L/min) with a fraction of inhalational oxygen (FIO_2_) of 0.4 was initiated due to worsening respiratory pattern. On the day HFNC oxygen therapy was initiated, his respiratory pattern temporarily improved but worsened again on the night of the 6th day of symptom onset. Chest radiography and CT revealed severe pneumomediastinum broadly extending to the pericardial and cervical regions, which was complicated by subcutaneous emphysema (Figs. 1 and 2A–C). He was transferred to our ICU with 10 L/min of supplemental oxygen administration on the 7th day.

After admission to the ICU, tracheal intubation was performed using 5 mg of rocuronium (a 3.5 mm ID cuffed oral endotracheal tube), and mechanical ventilation with pressure assist control was initiated. The PaO_2_/FIO_2_ ratio (P/F ratio) was 116, and the static lung compliance (Cstat) was 0.35 mL/cmH_2_O/kg with mechanical ventilation after endotracheal intubation using a 3.0-mm cuffed tube. Because of vigorous effort of spontaneous breathing and patient-ventilator asynchrony with an estimated driving pressure of 24 cmH_2_O according to tidal volume and Cstat, continuous infusion of rocuronium 7 μg/kg/min was initiated 50 minutes after intubation. The driving pressure was controlled at a low level to maintain the ventilation volume at <6 mL/kg and the plateau pressure at <28 cmH_2_O, with end-expiratory pressure of 5 cmH_2_O. The continuous infusion of muscle relaxant was discontinued after 42 hours, and spontaneous breathing was facilitated to prevent diaphragm atrophy. The P/F ratio gradually increased, and chest radiography findings showed improvement. CT revealed improvements of pneumomediastinum and subcutaneous emphysema (Fig. 2D–F). The P/F ratio improved to 361, and the Cstat was 0.76 mL/cmH_2_O/kg after controlling the fluid balance with loop diuretics. The tracheal tube was removed on the 7th ICU day. Immediately after extubation, HFNC oxygen therapy with 7 L/min (FIO_2_, 0.25) was reintroduced, but the patient was weaned off for a day owing to good oxygenation and a good respiratory pattern. Oxygenation was maintained within the normal range in room air, and he was discharged to the general ward on the 9th ICU day without any worsening of pneumomediastinum or mediastinitis.

## 3. Discussion

We presented a rare case of RSV infection with extensive pneumomediastinum and subcutaneous emphysema that was successfully managed with a lung-protective strategy using a muscle relaxant. To our knowledge, there is only 1 report on the requirement of tracheal intubation for pneumomediastinum associated with RSV infection in children.^[14]^ Kaneki et al classified the severity of spontaneous pneumomediastinum based on the development of subcutaneous emphysema and air space in the mediastinum on chest radiography (grades 0–4) and free air in the mediastinum on CT (grades A–C),^[15]^ which was graded as 3 and grade C, respectively, in the present case. The pathophysiological mechanism of pneumomediastinum is known as the Macklin effect,^[16]^ in which abrupt overinflation of the alveoli leads to rupture and the released alveolar air dissects through the pulmonary interstitium along the bronchovascular sheath toward the pulmonary hila, eventually reaching the mediastinum and causing pneumomediastinum.^[17,18]^ In the present case, lung overinflation and severe coughing induced an excessive increase in the intra-alveolar pressure, which may have resulted in pneumomediastinum. Frequent nasopharyngeal suctioning to clear the airway and increased airway pressure by HFNC oxygen therapy can induce pneumomediastinum, pneumothorax, and subcutaneous emphysema,^[19,20]^ thereby aggravating the patient’s respiratory condition. Invasive mechanical ventilation with a muscle relaxant reduced the work of breathing, driving pressure associated with excessive spontaneous breathing, and prevented the expansion of the pneumomediastinum via coughing and by patient-ventilator asynchrony. The severity of RSV infection typically peaks around 5 days after the onset of symptoms and improves within 7 to 10 days in most patients.^[3]^ Our patient was admitted to the ICU on the 7th day of RSV symptom onset. Lung-protective ventilation under neuromuscular blockade was implemented for approximately 2 days with the assumption that the symptoms would improve in 2 or 3 days, after which we started weaning from mechanical ventilation. The patient’s respiratory findings, including P/F ratio, Cstat, and CT image findings, improved as expected on the 5th ICU day.

## 4. Conclusion

We presented a rare case of an infant with severe pneumomediastinum associated with RSV infection who required mechanical ventilation. Lung-protective ventilation with muscle relaxants effectively prevented worsening of pneumomediastinum due to coughing and patient-ventilator asynchrony.

## Acknowledgments

The authors sincerely thank the patient and his family for providing permission to report this case.

## Author contributions

**Data curation:** Makiko Konda, Taichi Kotani.

**Supervision:** Masahiko Kawawguchi.

**Writing – original draft:** Junji Egawa.

**Writing – review & editing:** Yusuke Naito, Shota Sonobe.
